# Editorial: Emerging advancements in the carbonic anhydrase field

**DOI:** 10.3389/fmolb.2025.1624394

**Published:** 2025-05-22

**Authors:** Simona Maria Monti, Vincenzo Alterio, Seppo Parkkila, Claudiu T. Supuran, Giuseppina De Simone

**Affiliations:** ^1^ Institute of Biostructures and Bioimaging, Consiglio Nazionale delle Ricerche (IBB-CNR), Naples, Italy; ^2^ Faculty of Medicine and Health Technology, Tampere University, Tampere, Finland; ^3^ Fimlab Laboratories, Tampere University Hospital, Wellbeing Services County of Pirkanmaa, Tampere, Finland; ^4^ Department of NEUROFARBA, Section of Pharmaceutical and Nutraceutical Sciences, University of Florence, Firenze, Italy

**Keywords:** photosynthesis, hypoxia, vasculature, ferroptosis, tumour, tumor, drug design, dual targeting

Carbonic anhydrases (CAs; EC 4.2.1.1) are a superfamily of ubiquitous metalloenzymes that catalyze the reversible hydration of carbon dioxide to bicarbonate and proton, a reaction central to numerous physiological processes. CAs are phylogenetically classified into eight distinct families, α, β, γ, δ, ζ, η, ɵ, and ι, each characterized by unique structural motifs, quaternary arrangements, subcellular localizations, and kinetic parameters. These enzymes typically employ a Zn^2+^ ion at the catalytic core, although alternative metal cofactors such as Cd^2+^, Fe^2+^, and Co^2+^ have been identified in specific isoforms and environmental contexts.

CAs are versatile enzymes being involved in pH homeostasis, gas exchange, photosynthesis, lipogenesis, gluconeogenesis, and ureagenesis. In humans, dysregulation of CA expression or activity is implicated in a wide range of pathophysiological conditions, underscoring their relevance as therapeutic targets. Concurrently, the robustness and catalytic efficiency of CAs have positioned them as promising tools in biotechnological applications, including CO_2_ capture, biosensing, and synthetic biology.

Within this Research Topic we highlighted recent advances in CA research. The article carried out by Weerasooriya’s et al. Group investigates the synergistic roles of two carbonic anhydrase isoforms, αCA2 and βCA4.1, in the growth of *Arabidopsis thaliana* under varying CO_2_ conditions. Although plants are known to express multiple α- and β-type CAs, the specific contributions of individual isoforms within distinct subcellular compartments, and under non-ambient CO_2_ regimes, have remained unclear. Using fluorescent tags and GUS reporters, the authors show unambiguously that αCA2 resides in the cell wall while βCA4.1 anchors at the plasma membrane. The two mentioned CAs jointly sustain Arabidopsis growth only when CO_2_ falls to 200 μL L^-1^. Single knockouts show no defects at 200, 400 or 1,000 μL L^-1^ CO_2_, but the double mutant has reduced biomass and photosynthetic capacity at 200 μL L^-1^ rescued by reintroducing either isoform.

McDonald and Dedhar’s concise mini-review redefine CA IX not only as an important mediator of pH regulation in hypoxic tumor microenvironments but also as a pivotal regulator of ferroptotic susceptibility. Over the past decades, genetic, pharmacological, and *in silico* investigations have robustly established the therapeutic potential of targeting CA IX most notably with the selective CA IX/XII inhibitor SLC-0111, as a promising strategy against “difficult to treat” solid tumors. Yet adaptive resistance and tumor recurrence limit its long-term efficacy. Recent work here reviewed reveals that CA IX blockade unmasks metabolic co-vulnerabilities that sensitize cancer cells to cytotoxic agents by promoting ferroptosis regulated cell death that results from accumulation of toxic levels of phospholipid peroxidation. Authors suggest a co-targeting of CAIX/XII activity in combination with ferroptosis inducers to achieve substantial progresses in treating hypoxic tumors, especially those exhibiting chemo- and radio-resistance.

The review by García-Llorca et al., highlights the emerging role of CAs as key regulator of vascular tone, with CA inhibitors (CAIs), notably sulfonamides, provoking robust vasodilation in cerebral and retinal vessels. Although CAIs lower intraocular pressure and increase blood flow in select organs, their effects are organ-specific and the precise molecular mechanism remains elusive. It is likely that cytosolic CA isoforms are primarily involved, but it is still not clear which of them are most important. The understanding of which CA isoforms and downstream pathways drive this response could enable design of next-generation selective CAIs.

Finally, D’Ambrosio et al. reviewed from a structural point of view the development of novel therapeutics employing hCA inhibitors as dual-targeting compounds for the treatment of complex diseases. Authors shed light on the combined inhibition of hCAs, whose dysregulation is associated to a variety of human pathologies, with a second molecular target. Dual targeting may represent a promising way for developing more effective drugs. Successfully engineered hCA dual inhibitors could pioneer next-generation therapies for multifactorial disorders, enhancing therapeutic outcomes while reducing side effects and drug resistance, especially in complex conditions such as cancer, inflammation, and neurological disorders.

